# Tensile Behavior of Polyurethane Organic Polymer and Polypropylene Fiber-Reinforced Sand

**DOI:** 10.3390/polym10050499

**Published:** 2018-05-04

**Authors:** Jin Liu, Zhihao Chen, Zezhuo Song, Yuxia Bai, Wei Qian, Jihong Wei, Debi Prasanna Kanungo

**Affiliations:** 1 School of Earth Sciences and Engineering, Hohai University, Nanjing 210098, China; hhuczh@163.com (Z.C.); szzhhu@163.com (Z.S.); byxhhu@163.com (Y.B.); wei.geoserve@gmail.com (W.Q.); weijhhhu@163.com (J.W.); 2 CSIR-Central Building Research Institute (CBRI), Roorkee 247667, India; debi.kanungo@gmail.com

**Keywords:** geosynthetics, polymers, composite reinforcement, tensile strength, interaction mechanism, dry density

## Abstract

Physical and chemical reinforcements are commonly used to improve sand properties for engineering requirements. Many researchers have concluded that composite reinforcement can greatly improve sand property strength. In this paper, polyurethane organic polymer (PU) and polypropylene fiber (PF) were used to reinforce sand. It is found that composite reinforcement has great effects on tensile strength. A series of direct tensile tests were conducted to demonstrate this reinforcement and to investigate the effects of PF content, PU content, dry density, and curing time. Additionally, the reinforcement mechanism was analyzed by scanning electron microscope images. The tensile strength increases with curing time until it reaches a plateau. The composite reinforcement improves the tensile strength exponentially with the increase of PF and PU contents. For the effect due to dry density, the tensile strength first increased and then decreased with the peak at approximately 1.55 g/cm^3^. Through the interaction force among fibers and sand particles and the bonding force of polymer among sand particles, tensile strength of reinforced sand is greatly improved.

## 1. Introduction

Sand causes all kinds of geological engineering problems due to its instability. Most natural sand needs reinforcement to meet engineering requirements. Sand reinforcement can be physical and/or chemical. For physical reinforcement, one common stabilization method is to add various reinforcement materials into the sand, such as geogrid, geotextile, fiber, and so on. Fiber reinforcement is considered as an easy and effective method when discrete and randomly distributed fibers are included because of convenient mixing procedure, good strength properties, and ecologic potential. Many researchers have investigated fiber-reinforced soil [1,2,3,4] and found that the mechanical properties of soils can be improved strongly by including fibers. For chemical reinforcement, several studies have shown that cement or fly ash can lead to greater shear and compressive strength [5,6,7,8]. It also creates better control of displacement and prevents failure. However, most chemical reinforcements will have negative effects on the environment in an irrecoverable way. The concept of reinforcing sand using polymeric materials has attracted growing attention in engineering applications. Many experiments have confirmed that polymers benefit the strength of both clay and sand [9,10,11,12,13]. This strategy might cause air and water pollution because of volatile organic compounds, residual polymer solution, and microplastic pollution, but the environmental impact of polymeric reinforcement can be minimized under control. As expected, only fibers are not enough to make sand shaped for reinforcement. Therefore, polymers are introduced to make the sand form a unitary coherent matrix. The combination of physical and chemical reinforcement can create a synergetic effect [14,15,16,17,18,19]. Our polymer-fiber mixture is a step in this direction.

In this paper, we focus on tensile strength. Tensile strength has not received the attention it deserves, even though it is closely related to soil cracking. A few studies have reported reinforcement effects on the tensile strength [20,21,22,23], but insufficient research has been done on composite reinforcement. The research that has been done mostly deals with clayey soil. However, since sand has been reinforced by PF and PU, the tensile strength may be examined.

This study aims at investigating the tensile behavior of polymer-fiber reinforced sand for optimum results in terms of PF content, PU content, dry density, and curing time. A series of tensile tests were conducted to quantify the tensile strength of reinforced sand specimens. Scanning electron microscope images were also used to investigate the microstructure of specimens to shed light on the composite reinforcement mechanism.

## 2. Materials and Methods

### 2.1. Materials

#### 2.1.1. Sand

**Figure 1:**
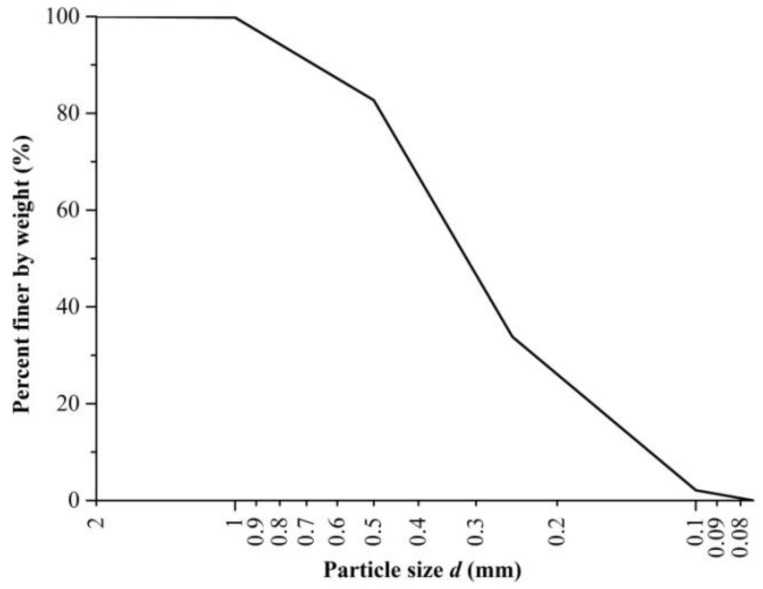
Grain size distribution of sand.

The sand used in this study was collected from Nanjing area, eastern China. The sand was air-dried, crushed, and sieved at 2 mm in the laboratory. Its physical properties are shown in Table 1. The grain size distribution of sand is presented in Figure 1. It can be seen that the sand particles have an effective grain size (d_10_) of about 0.14 mm, graduation coefficient (C_c_) of 1.13, and uniformity coefficient (C_u_) of 2.77. The size of 0.1~0.5 mm particles is about 80%. 

#### 2.1.2. Polypropylene Fiber

Polypropylene fiber (PF) is used as reinforcement material in this study. It has a length of 18 mm and diameter of 0.034 mm. It has high strength, good elasticity, and excellent dispersibility. Its basic physical parameters are given in Table 2.

#### 2.1.3. Polyurethane Organic Polymer

**Figure 2:**
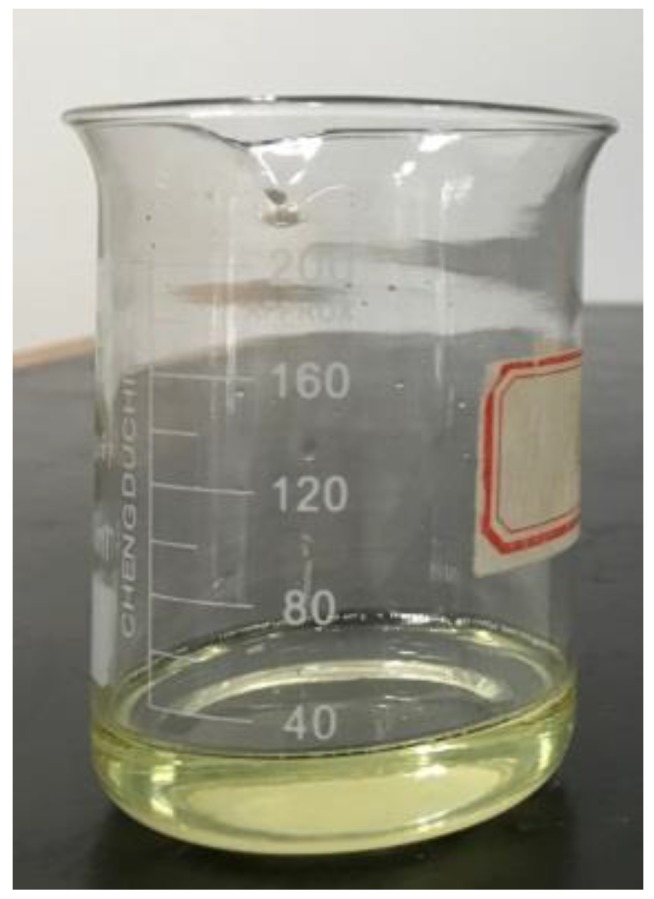
Photograph of polyurethane organic polymer.

Polyurethane organic polymer (PU) shown in Figure 2 is used in this study, and its properties are listed in Table 3. It has a main constituent of polyurethane resin and contains enormous amount of isocyanate groups (-NCO). This kind of functional group allows pre-polymer to react with water rapidly and thus produce polyurea.

The synthesis of polyurethane prepolymer is as follows. The polyurethane prepolymer were prepared by one-step process based on Poly-oxyPropylene Glycol (PPG) with molecular weight about 1000, Poly-oxyEthylene Glycol (PEG) with molecular weight about 1000, Poly-CaproLaclone glycol (PCL) with molecular weight 3000, and toluene. These raw materials were prepared by dehydration treatment. Azeotrope distillation method was adopted to remove trace water in polyols. Polymer polyols and toluene were first mixed at 130 °C. Air distillation was adopted until toluene was close to steaming, and then the vacuum distillation was adopted to eliminate residual water and toluene in the reaction system. After that, the temperature was cooled to 20 °C, a reflux condensing tube was placed, and the reaction system was sealed with oil. Toluene diisocynate (TDI) was added and stirred for two hours at 100 °C. The reaction system should be in the nitrogen, and polyurethane prepolymer was produced by polycondensation. Subsequently, the reaction system was cooled down to room temperature, and ethyl acetate was mixed with the prepolymer for one hour. Prior to obtaining the desired product, sodium dodecyl benzene sulfonate (SDBS) was added as surfactants, and polyurethane organic polymer was obtained after thorough stirring.

### 2.2. Experimental Methods

The specimens were prepared by hand mixing sand, fibers, polymers, and water. To investigate the effects of PF and PU content, four PF contents (*f* = 0.1, 0.2, 0.3, and 0.4% by weight of dry sand) and four PU contents (*p* = 1, 2, 3, and 4% by weight of dry sand) are used in the experiments. Five dry densities ($\rho_{d}$) of 1.40, 1.45, 1.50, 1.55, and 1.60 g/cm^3^ are studied. The curing times (*t*) are set to 6, 12, 24, 48, 72, and 96 h, and the effect of curing time under room temperatures is investigated. The water content was controlled to about 10% when specimens were just finished.

In the experiments, fibers were first mixed with dry sand in small increments manually. After the initial mixing of the two, a mixture of polymers and water was added into sand. Thorough mixing is essential here to obtain a homogeneous product. Prior to the solidification of PU, the mixture was transferred to the mold and compacted to the target density. Before tensile tests, the prepared mixtures were conserved under room temperature for PU to completely solidify.

**Figure 3:**
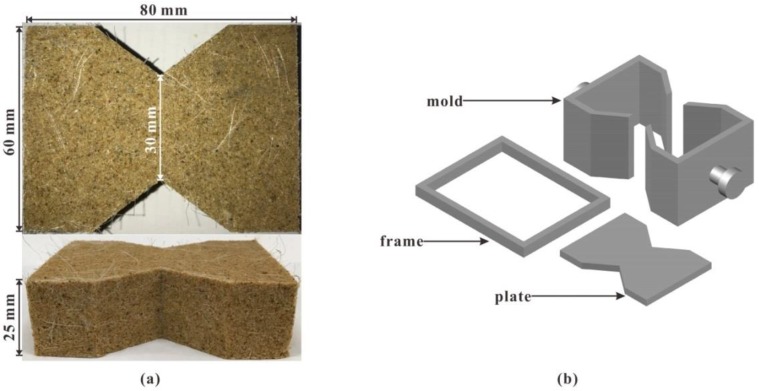
Schematic drawing of (**a**) prepared specimen and (**b**) mold and its components.

**Figure 4:**
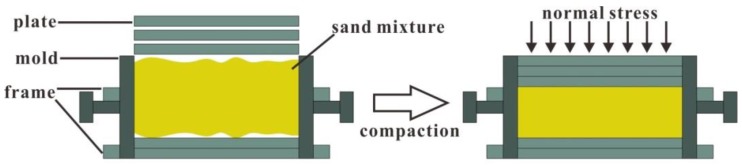
The compaction process of a specimen.

Figure 3a shows the prepared specimen and its dimensions. The specimen is wide on both sides and narrow in the middle. It is 80 mm long, 60 mm wide, and 25 mm high. The specimen width is reduced from 60 to 30 mm to make the tensile failure take place in the central section. Figure 3b shows the mold (50 mm high), which is designed to prepare specimens and perform tensile tests. The mold consists of two halves of the split mold with its interior space to hold the specimen. During compaction, two rectangular metal frames were used to restrict the movement of the mold, and several iron plates of 5 mm thick were deployed to control the compaction height, as shown in Figure 4. In this way, the density of a specimen can be controlled by compaction height. The sand mixture was put in the mold, where three iron plates were placed above it and the other two were placed underneath. The sand mixture would not spill out, as there was enough space in the mold. Then, the sand mixture was compacted to the target height (25 mm) with a jack. Note that the top iron plate is on the same height as the top of the mold after the compaction.

**Figure 5:**
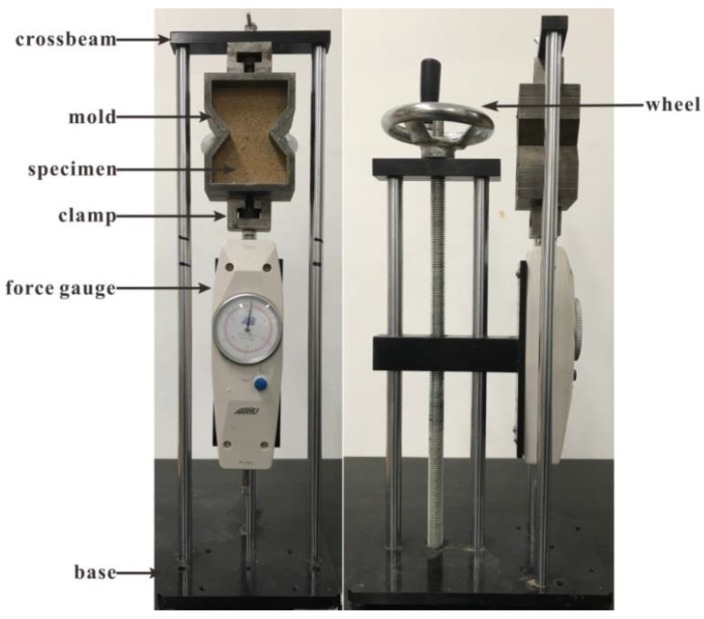
Photograph of the tensile test apparatus.

We perform a direct tensile test to investigate the reinforced sand. A pulling force is applied directly on the specimen until tensile failure in the middle section. After compaction, the specimen was extracted from the mold and then conserved for certain time. After curing time, the specimen was put into the mold. The mold with the specimen was then transferred to a tensile test apparatus (Figure 5). The upper clamp is fixed on the crossbeam, and the lower clamp is connected to a force gauge that has a capacity of 300 N and an accuracy of 0.1 N. The distance between the upper and lower clamps is adjusted to fit in the mold. The lower part of the mold is connected to the force gauge through a clamp, and the upper part of the mold is clamped to the crossbeam. A rotating wheel is used to drive the force gauge to move downward vertically at a constant speed. This results in a tensile force being applied directly on the specimen. When the specimen was broken, the maximum tensile force was recorded by the force gauge, and the tensile strength was calculated using the following equation:

(1) $$\sigma_{t}=\frac{2T+W_{1}+W_{2}}{2S}$$

in which T (N) = maximum tensile force, $W_{1\;}$(N) = specimen weight, $W_{2\;}$(N) = mold weight, S (m^2^) = cross-sectional area (0.030 m × 0.025 m), and $\sigma_{t}$ (kPa) = tensile strength.

At least five parallel specimens are prepared for each tensile strength, and the average value is taken as a result. To minimize the experimental error, it is required that valid individual tensile strength be within 10% of the mean value.

## 3. Results and Discussion

### 3.1. Tensile Test Results

The tensile strength of reinforced sand was measured under different PF content, PU content (A1–A4), dry density (B1–B3), and curing time (C1–C3). A total of 69 groups of tensile tests were conducted, and the results are listed in Table 4, Table 5 and Table 6.

### 3.2. Effect of Curing Time

**Figure 6:**
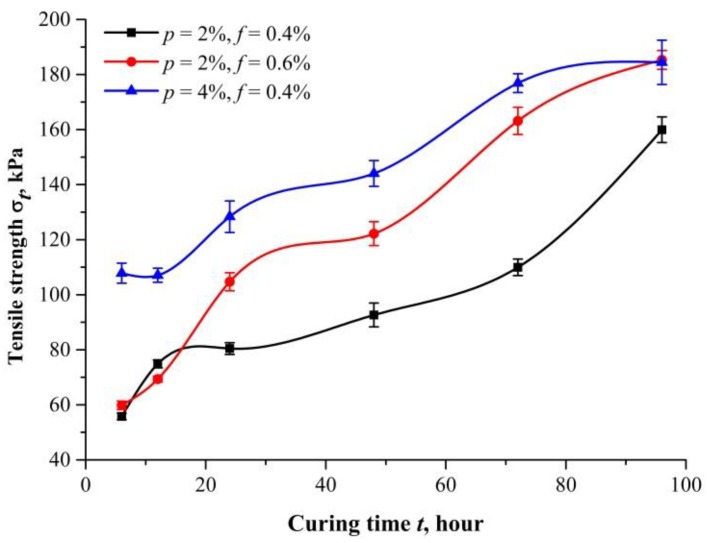
Tensile strength of specimens with different curing times. The bars represent standard deviations.

Figure 6 shows the tensile strength at different curing times (*t* = 6, 12, 24, 48, 72, and 96 h) and its error bars. Three proportions (*p* = 2%, *f* = 0.4%; *p* = 2%, *f* = 0.6%; *p* = 4%, *f* = 0.4%) are investigated with all specimens compacted at 1.50 g/cm^3^ dry density. The tensile strength grows over time and tends to saturate at the value shown in Figure 6. This process of gradual increase in tensile strength is similar to cement hydration, because PU needs enough time to condense and harden. Regardless of PF content, the tensile strength of specimens with the same PU content is almost same in the first few hours of curing (from 6 to 12 h). The benefits of fiber reinforcement can only be observed until PU has condensed. By contrast, the specimens with higher PU content had a greater tensile strength regardless of curing time. This indicates that the bonding force produced by PU is essential for tensile strength.

The effects of curing time have two folds, fiber reinforcement benefit, and the role of PU. The former contributes to the interfacial shear strength and the later bonds the sand particles together to fibers. An increase in water content leads to a tensile strength reduction. This is due to the fact that increased lubrication makes sand particles more easily driven by PF and rearrange under a tensile load. Many studies have shown that the tensile strength of natural soils generally decreases with the increase of water content [24,25,26,27]. Tang et al. [28] measured the interfacial shear strength of fiber-reinforced soil and found that it decreases as water content increases. When the water content is relatively high, the bonding and interlock force decreases, resulting in the weakened pull-out resistance of fibers. This explains why the tensile strength of specimens with different PF contents is almost identical at small curing time (*t* = 6 and 12 h), as shown in Figure 6. Polymer reinforcement is controlled by the quantity and curing time of polymeric membrane that immobilizes fibers and sand particles. As curing time increases, stronger cohesion is provided. For curing time of 96 h, the tensile strength of specimen with 2% PU and 0.6% PF is close to that with 4% PU and 0.4% PF. This suggests that fibers play an important role in long-term strength. However, further research is necessary to determine whether PU will crack due to aging that leads to a decrease of tensile strength. Based on previous studies by Liu et al. [29] for polyurethane organic polymer reinforced sand, the unconfined compressive strength plateaus were at about 48 h. A similar curing time is therefore chosen to be investigated.

### 3.3. Effect of PF Content

**Figure 7:**
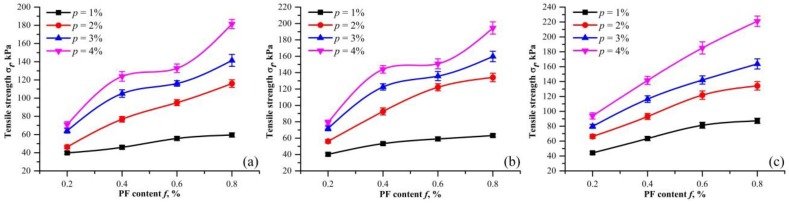
Tensile strength of specimens with different PF content at different dry density: (**a**) 1.40 g/cm^3^; (**b**) 1.50 g/cm^3^; (**c**) 1.60 g/cm^3^ (*t* = 48 h, and the bars are standard deviations).

Figure 7 shows the relationship between the tensile strength and different PF content for three dry densities ($\rho_{d}=$ 1.40, 1.50 and 1.60 g/cm^3^). The error bars from the measurement are presented as well. The tensile strength increases monotonically with PF content. For dry density of 1.50 g/cm^3^, the tensile strength increased from 56.13 to 134.12 kPa, when PF content increased from 0.2% to 0.8% with 2% fixed PU content. For dry density of 1.60 g/cm^3^, the tensile strength increases from 66.27 to 134.23 kPa under the same condition. This increase demonstrates the effectiveness of PF in sand reinforcement.

At a certain density, Figure 7 also shows that the tensile strength increases more rapidly with PF content for PU content of 2, 3, and 4%. This clearly demonstrates the synergy of PF and PU at late curing times. Fiber reinforcement is determined by the bonding strength and friction between fibers and soil particles [28]. For unreinforced sand, its loose structure cannot take the strength of PF. A cohesive force provided by PU is needed for PF to function. This explains the difference in PF effect with the various PU contents. Moreover, with the increase of dry density, the tensile strength improvements with respect to PF content changes from ladder-like to roughly a straight line. The reason is that since the elastic stiffness of fibers is low, a slight relative displacement between fibers and sand particles is not sufficient to mobilize the frictional resistance [30]. The same is found in the tensile tests that the embedded fibers have a greater tendency to be pulled out rather than elongated and broken for low dry density and PU content.

**Figure 8:**
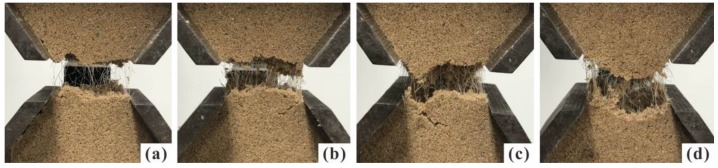
Photographs of specimen with different PF content after test: (**a**) *f* = 0.2%; (**b**) *f* = 0.4%; (**c**) *f* = 0.6%; (**d**) *f* = 0.8% (*p* = 2%, $\rho_{d}$ = 1.50 g/cm^3^ and *t* = 48 h).

Fibers served as bridges that share the tensile stress when the tension cracks were developing. These fibers effectively impede the further development of tensile cracks. As observed in Figure 8, the fiber benefits became more obvious with the increase of PF content. At higher PF content, fibers have more chances to present in a fracture. This increases the ability to bear large tensile load. At tensile failure, the displacement concentrates on the extension of fibers, and fiber breakage may occur when tensile load exceeds the tensile strength of fibers [31,32]. As a result, the specimen performed a ductile behavior. Previous researches also showed that fiber inclusion improves the compression strength and ductility of soil [16,33,34]. It is considered that the anchoring effect of sand matrix on fibers can give full play to the ductility of fibers.

### 3.4. Effect of PU Content

**Figure 9:**
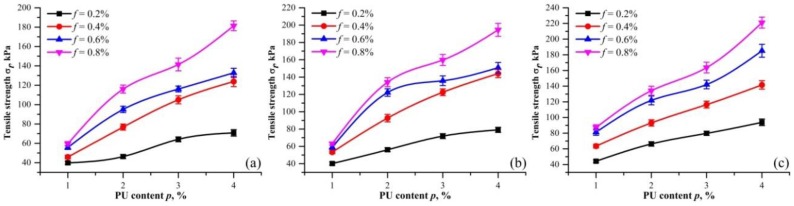
Tensile strength of specimens with different PU contents at different dry densities: (**a**) 1.40 g/cm^3^; (**b**) 1.50 g/cm^3^; and (**c**) 1.60 g/cm^3^ (*t* = 48 h, and the bars are standard deviations).

Figure 9 shows the relationship between the tensile strength and different PU content for three dry densities ($\rho_{d}=$ 1.40, 1.50, and 1.60 g/cm^3^). The error bars from the measurement are also presented. For fixed dry density and PF content, the tensile strength increases with PU content. For instance, the tensile strength for 0.4% PF content and 1.50 g/cm^3^ dry density increases from 53.32 to 144.05 kPa when the PU content increases from 1% to 4%. For the same PU content, the tensile strength again presented an increase with PF content. This, once again, shows the synergy effect of PF and PU. By contrast, the tensile strength curve in Figure 9c is more regular. It is considered that compaction was the main cause of this appearance. Under stronger compaction, PU can be more evenly distributed in specimens.

**Figure 10:**
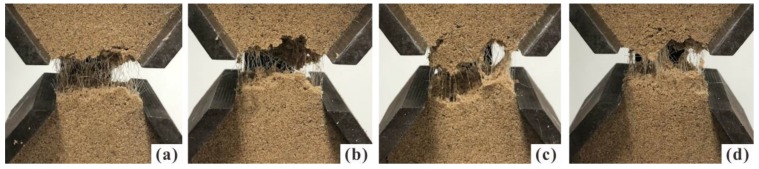
Photographs of specimen with different PU content after test: (**a**) *p* = 1%; (**b**) *p* = 2%; (**c**) *p* = 3%; and (**d**) *p* = 4% (*f* = 0.4%, $\rho_{d}$ = 1.50 g/cm^3^ and *t* = 48 h).

An increase in PU content can also lead to failure form change. The states of specimens with different PU contents during tensile tests are illustrated in Figure 10. The failure form gradually changes from brittle to plastic as PU content increases. In addition to the extension of fibers, polymeric membrane can also efficiently impede the further opening and development of tensile cracks. This protects specimens from complete brittle failure. Several studies showed that the cohesive force and frictional angle can be improved significantly by polymers [35,36]. Therefore, the beneficial effects of polymer reinforcement on tensile strength can be attributed to the increase of bonding force between sand particles and the interfacial force between fibers and sand particles. With an increase of PU content, fibers are more closely bonded with the sand particles, thereby the interface forces increase with the contact area. In comparison of Figure 10a,d, more polymeric membranes can be observed linking the fracture.

### 3.5. Effect of Dry Density

**Figure 11:**
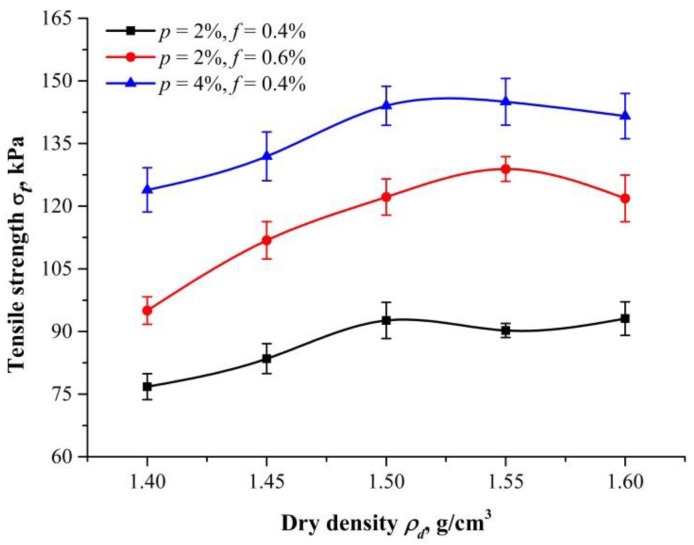
Tensile strength of specimens with different dry density (*t* = 48 h, and the bars are standard deviations).

Figure 11 shows the tensile strength and its error bars at different dry densities ($\rho_{d}=$ 1.40, 1.45, 1.50, 1.55, and 1.60 g/cm^3^). Three proportions (*p* = 2%, *f* = 0.4%; *p* = 2%, *f* = 0.6%; and *p* = 4%, *f* = 0.4%) are used for this investigation. It is observed that tensile strength reaches maximum at about 1.55 g/cm^3^ dry density. For larger or smaller dry densities, the tensile strength will become less. This finding is consistent with that of Liu et al. [37]. For 2% PU and 0.6% PF contents, the tensile strength increases from 95.00 to 128.90 kPa for dry density increases from 1.40 to 1.55 g/cm^3^. It decreases from 128.90 to 121.86 kPa for dry density increases from 1.55 to 1.60 g/cm^3^, as shown in Table 4.

**Figure 12:**
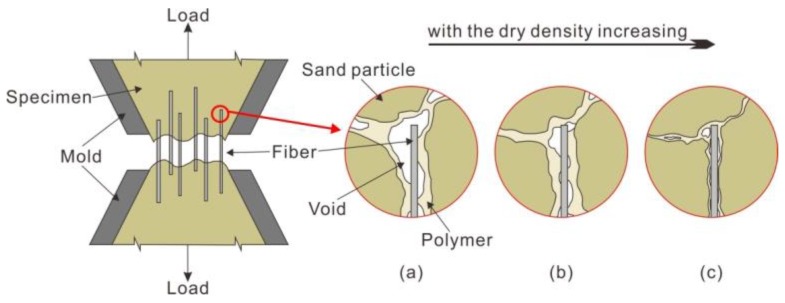
Schematic drawing of microstructure of specimens at dry densities of (**a**) 1.40 g/cm^3^; (**b**) 1.50 g/cm^3^; and (**c**) 1.60 g/cm^3^.

For a better understanding of the effect of dry density, a schematic drawing is presented in Figure 12 for illustration. Tensile strength variation with dry densities is caused by contact conditions. For specimens with a small dry density, the voids between sand particles are relatively large, resulting in insufficient filling of PU. Therefore, the polymeric membrane tends to enwrap sand particles and fibers instead of connecting (Figure 12a). It is limited by disadvantages of weak connecting forces, which limit the increase in tensile strength. As dry density increases, more contacts are created, and the membrane will contribute more to connecting forces and interfacial mechanical interaction (Figure 12b). All these benefits prevent embedded fibers from being pulled out, resulting in even significant fiber reinforcement. This explains the increase of tensile strength, as dry density increases before 1.55 g/cm^3^. However, large compactions are needed in preparation when specimen has a higher dry density. This leads to the membrane wrapped around sand particles and fibers becoming thinner, and the connecting force between sand particles becomes weaker. Thus, the displacement of sand particles is less restricted (Figure 12c). This explains the decrease of tensile strength with increase of dry density beyond 1.55 g/cm^3^. Therefore, a high dry density has a negative effect on the tensile strength of specimens instead.

### 3.6. Tensile Curves

**Figure 13:**
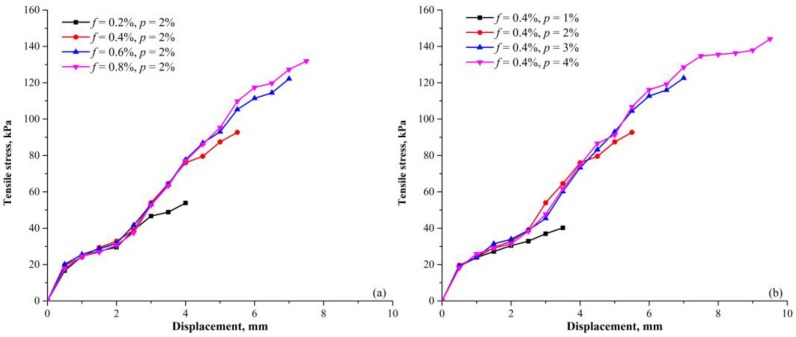
The typical tensile curves of specimens with different (**a**) PF content and (**b**) PU content (*t* = 48 h and $\rho_{d}$ = 1.50 g/cm^3^).

Figure 13a shows the typical tensile curves (tensile strength versus displacement) of the specimens with different PF contents. The tensile stress increases monotonically with the increase of displacement before cracking. The increase rate is basically constant, but it gradually decreases when approaching the peak. In Figure 13b, the typical tensile curves with different PU contents are shown. The increase rate is not influenced by PF and PU contents. Instead, the presence of PF and PU improves the ductility of specimen significantly, resulting in larger displacement before the peak is reached. Based on previous studies [21,38], it is expected that the residual tensile strength can be observed after cracking rather than the tensile stress drops to zero abruptly. This is because further displacement will be concentrated on elastic extension of fibers and polymeric membranes across the opening section once tensile cracks are formed (Figure 8 and Figure 10).

## 4. Composite Reinforcement Mechanism

The PU reaction process is given in formulas (2) and (3). Generally, PU has no chemical reaction with sand particles. Instead, the two are mainly bonded by intermolecular force and hydrogen. The effective groups on the long chain can be adsorbed on the sand particle surfaces to form a thin and tough membrane.

(2) $$O=C=N-R-N=C=O+2H_{2}O\to \mathrm{HO}-\mathrm{CO}-\mathrm{NH}-R-\mathrm{NH}-\mathrm{CO}-\mathrm{OH}\to H_{2}N-R-{\mathrm{NH}}_{2}+2{\mathrm{CO}}_{2}$$

(3) $$\left(n+1\right){\;H}_{2}N-R-{\mathrm{NH}}_{2}+n\;O=C=N-R-N=C=O\to H_{2}N\;{\left[R-\mathrm{NH}-\mathrm{CO}-\mathrm{NH}\right]}_{2n}\;R-{\mathrm{NH}}_{2}$$

**Figure 14:**
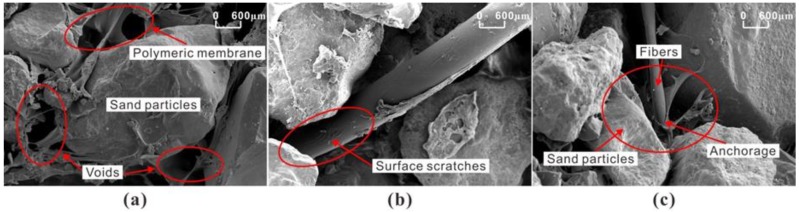
SEM images of 200 times magnification: (**a**) space network structure; (**b**) fiber surface scratches; and (**c**) anchorage effects.

The reinforcement mechanism of PU can be categorized into three parts: enwrapping, filling, and coupling. At preparation, the polymer solution was fully mixed with sand, and it formed an elastic and viscous membrane to wrap sand particles and fibers. During compaction, the unsolidified polymer solution can fill the voids and connect adjacent sand particles and fibers. A space network membrane structure forms, as shown in Figure 14a. This membrane structure increases the bonding and interlocking forces between sand particles, which glues the loose sand and fibers as an integrated whole. Moreover, this polymeric membrane is a lightweight flexible material composing a skeletal matrix of tiny interconnecting strands that act as an effective energy transfer path. It is expected that the tensile force can be evenly distributed along the failure section due to the stability of this membrane structure. With the evaporation of water, the contraction and tension of membrane surface produces a deformation that is tensile stress-resistant.

It is also found that bonding strength and friction play a dominant role in fiber reinforcement. When fibers are uniformly distributed in the sample, they cross and overlap to form a network to restrain displacement of sand particles. After the tensile test, it is observed that the fiber surface has obvious scratches, as shown in Figure 14b. This suggests strong frictions between fibers and sand particles, indicating an outward relative sliding of fibers must have occurred during tensile test. The interfacial shear resistance contributed by the bonding strength and friction can restrict the relative movement of fibers and prevent fibers from being pulled out. The frictional force is largely determined by the roughness and tightness of interfaces between fibers and sand particles, which can also effectively counteract the interfacial shear force. Several researchers have found that the fiber sliding resistance is strongly dependent on the fiber surface roughness [39,40]. With PF content and dry density increasing, the effective contact area between fibers and sand particles will increase. This leads to the enhancement of the friction force, and thereby more constraints on displacement of the sand particles and fibers. As shown in Figure 14c, there is an anchorage which makes the fiber difficult to pull. Fibers are more likely to have elastic deformation under the tensile force in this situation. As a result, fibers can bear larger load and distribute the stress to a broader area. However, excessive content of fiber will easily gather into clusters, making the polymeric membrane difficult to enwrap and forming a stable structure [41]. This leads to a weak coupling between sand particles and fibers. Therefore, over supply of fibers may cause a decline in tensile strength. Further studies are needed to quantify this critical PF content.

Due to the fact that the aging degradation of PU and PF is mainly controlled by temperature, ultraviolet ray, water, and chemical mediator, it is ideal to apply this enforcement under the conditions of temperature −10~50 °C, ultraviolet intensity ≤ 250 mW/m^2^, and no chemical mediator.

## 5. Conclusions

A series of tensile tests was conducted to study the tensile strength of PU and PF reinforced sand. The effects of PF content, PU content, dry density, and curing time on tensile strength are thoroughly investigated. SEM images are taken to understand the composite reinforcement mechanism. The conclusions are drawn as follows:

(1) The tensile strength is significantly influenced by curing time. With an increase in curing time, the tensile strength will increase gradually until saturation. The formed polymeric membrane is the prerequisite for the functioning of fibers. Owing to the presence of PF, the long-term strength can be improved.
(2) Composite reinforcement significantly improves the tensile strength of sand. The tensile strength increased monotonously with the increase of PF and PU content within our test range. The bonding force produced by PU and the connecting effect of PF are important factors for tensile strength improvement.
(3) It is found that there is an optimal dry density (around 1.55 g/cm^3^) where the tensile strength has an optimum response to reinforcement. At higher and lower dry densities, the tensile strength is less at the same reinforcement conditions.
(4) The PU reinforcement is generated by a spatial network membrane structure. The effects of the polymeric membrane are categorized as enwrapping, filling, and connecting. The PF reinforcement can be attributed to the interfacial force between fibers and sand particles. This force effectively prevents sand particles from rearranging under load.

## Figures and Tables

**Table 1:** Physical Properties of Sand.

Sand Properties	Value
Specific gravity (g/cm^3^)	2.65
Natural water content (%)	2
Maximum dry density (g/cm^3^)	1.66
Minimum dry density (g/cm^3^)	1.34
Maximum void ratio	0.970
Minimum void ratio	0.590

**Table 2:** Properties of polypropylene fiber.

PF Properties	Value
Density (g/cm3)	0.91
Average diameter (mm)	0.034
Average length (mm)	18
Breaking tensile strength (MPa)	300
Modulus of elasticity (MPa)	3500

**Table 3:** Properties of polyurethane organic polymer.

PU Properties	Value
Specific gravity (g/cm^3^)	1.18
Viscosity (MPa·s)	650~700
Appearance	Light-yellow transparent liquid
Mass fraction (%)	85
Solidification time (s)	30~1800
Water retention	Very good
pH	7

**Table 4:** Tensile strength of A1–A4 groups.

Test Number	PF Content *f* (%)	PU Content *p* (%)	Tensile Strength (kPa)/Standard Deviation (kPa)		
			$\rho_{d}=$ 1.40 g/cm^3^	$\rho_{d}=$ 1.50 g/cm^3^	$\rho_{d}=$ 1.60 g/cm^3^
A1-1	0.2	1	39.84/1.58	40.21/1.81	44.24/1.77
A1-2	0.2	2	46.32/2.03	56.13/2.28	66.27/2.87
A1-3	0.2	3	64.11/2.54	71.83/2.85	79.81/2.69
A1-4	0.2	4	70.78/3.32	79.06/2.98	93.85/4.19
A2-1	0.4	1	45.87/1.94	53.32/1.78	63.50/2.50
A2-2	0.4	2	76.78/3.11	92.64/4.33	93.09/4.01
A2-3	0.4	3	105.00/4.13	122.52/3.86	116.47/4.43
A2-4	0.4	4	123.90/5.30	144.05/4.67	141.57/5.42
A3-1	0.6	1	55.66/2.02	59.00/2.42	81.28/3.97
A3-2	0.6	2	95.00/3.28	122.19/4.34	121.86/5.59
A3-3	0.6	3	116.12/3.07	135.83/5.61	142.15/5.50
A3-4	0.6	4	132.80/4.71	150.81/6.11	185.15/8.19
A4-1	0.8	1	59.45/2.28	63.16/1.90	87.19/3.44
A4-2	0.8	2	116.04/4.15	134.12/5.18	134.23/5.72
A4-3	0.8	3	141.45/6.57	159.77/6.33	163.73/6.82
A4-4	0.8	4	181.47/5.05	194.51/7.48	221.03/6.98

**Table 5:** Tensile strength of B1–B3 groups.

Test Number	PF Content *f* (%)	PU Content *p* (%)	Tensile Strength (kPa)/Standard Deviation (kPa)	
			$\rho_{d}=$ 1.45 g/cm^3^	$\rho_{d}=$ 1.55 g/cm^3^
B1	0.4	2	84.49/3.57	90.23/1.69
B2	0.4	4	131.94/5.84	144.99/5.97
B3	0.6	2	111.82/4.46	128.90/5.60

**Table 6:** Tensile strength of C1–C3 groups.

Test Number	PF Content *f* (%)	PU Content *p* (%)	Tensile Strength (kPa)/Standard Deviation (kPa)					
			t=6	t=12	t=24	t=48	t=72	t=96
C1	0.4	2	55.80/1.20	74.88/1.44	80.44/2.12	92.64/4.33	109.96/2.98	159.94/4.64
C2	0.4	4	107.81/3.63	107.07/2.55	128.34/5.72	144.05/4.67	176.88/3.42	184.42/8.02
C3	0.6	2	59.79/1.55	69.35/1.08	104.71/3.39	122.19/4.34	163.16/4.94	185.29/3.41
